# Robot-assisted stereotactic brain biopsy: systematic review and bibliometric analysis

**DOI:** 10.1007/s00381-018-3821-y

**Published:** 2018-05-10

**Authors:** Hani J. Marcus, Vejay N. Vakharia, Sebastien Ourselin, John Duncan, Martin Tisdall, Kristian Aquilina

**Affiliations:** 1 Department of Neurosurgery, Great Ormond Street Hospital, London, UK; 2 Wellcome EPSRC Centre for Interventional and Surgical Sciences, University College London, 8.02 Malet Place Building, Gower Street, London, WC1E 6BT UK; 3 UCL Institute of Neurology, National Hospital for Neurology and Neurosurgery, Queen Square, London, WC1N 3BG UK

**Keywords:** Surgery, Robotics, Stereotaxy, Diffuse intrinsic brainstem glioma, DIPG

## Abstract

**Introduction:**

Stereotactic brain biopsy represents one of the earliest applications of surgical robotics. The aim of the present systematic review and bibliometric analysis was to evaluate the literature supporting robot-assisted brain biopsy and the extent to which the scientific community has accepted the technique.

**Methods:**

The Cochrane and PubMed databases were searched over a 30-year period between 1st of January 1988 and 31st of December 2017. Titles and abstracts were screened to identify publications that met the following criteria: (1) featured patients with brain pathology, (2) undergoing stereotactic brain biopsy, (3) reporting robot-assisted surgery, and (4) outcome data were provided. The reference lists of selected studies were also sought, and expert opinion sought to identify further eligible publications. Selected manuscripts were then reviewed, and data extracted on effectiveness and safety. The status of scientific community acceptance was determined using a progressive scholarly acceptance analysis.

**Results:**

All identified studies were non-randomised, including 1 retrospective cohort study and 14 case series or reports. The diagnostic biopsy rate varied from 75 to 100%, and the average target accuracy varied from 0.9 to 4.5 mm. Use of the robot was aborted in two operations owing to geometric inaccessibility and an error in image registration but no associated adverse events were reported. A compounding progressive scholarly acceptance analysis suggested a trend towards acceptance of the technique by the scientific community.

**Conclusions:**

In conclusion, robot-assisted stereotactic brain biopsy is an increasingly mainstream tool in the neurosurgical armamentarium. Further evaluation should proceed along the IDEAL framework with research databases and comparative trials.

## Introduction

Surgical robotics is amongst the most important technologies to emerge over the last decade [1]. Surgical robots may result in higher accuracy and precision than would otherwise be possible. The clinical corollary is that such robots may ultimately improve the safety and effectiveness of surgical interventions.

Stereotactic brain biopsy represents one of the earliest applications of surgical robotics. On the 11th of April 1985, a team at the Memorial Medical Center used a modified PUMA industrial robot (Advance Research & Robotics, CT, USA) to perform a robot-assisted stereotactic brain biopsy in a 52-year-old man [2]. Since this initial report, many surgical robots have been used to perform stereotactic brain biopsy including the Neuromate (Renishaw, Gloucestershire, UK), ROSA (Medtech, Montpellier, France), and Renaissance (Mazor Robotics, Caesarea, Israel) robots. Anecdotally, robot-assisted stereotactic brain biopsy has been adopted within the neurosurgical community.

Over recent years, considerable emphasis has been placed on the methodology of translation of new devices such as surgical robots from the laboratory to the operating room, the central tenet being that innovation and evaluation can, and should, proceed together in an ordered and logical manner [3–5]. The aim of the present systematic review and bibliometric analysis was to evaluate the literature supporting robot-assisted brain biopsy and the extent to which the scientific community has accepted the technique.

## Methods

The study protocol was registered on the international prospective register of systematic reviews (PROSPERO CRD42017082204). The Preferred Reporting Items for Systematic Reviews and Meta-Analyses (PRISMA) Statement was used in the preparation of this manuscript [6].

### Search methods

Two authors (HJM and VNV) independently searched the Cochrane Central Register of Controlled Trials (CENTRAL) and PubMed databases over a 30-year period between 1st of January 1988 (the year of the first publication) and 31st of December 2017. Search terms were generated with the PICO tool (Problem, Intervention, Comparison, and Outcome) and the Boolean free-text search [(brain OR brainstem OR cerebral OR cerebellar) AND (biopsy OR biopsies) AND (robot OR robotic)] used. The last date of the search was undertaken on the 4th of January 2018. The reference lists of selected studies were also sought, and expert opinion sought to identify further eligible publications. Duplicates were then removed and an English language restriction applied.

### Eligibility criteria

Titles and abstracts were screened to identify publications that met the following criteria: (1) featured patients with brain pathology, (2) undergoing stereotactic brain biopsy, (3) reporting robot-assisted surgery, and (4) outcome data were provided. Full publications were then obtained and assessed for eligibility. Any discrepancies were resolved by consensus and discussion with the senior author.

### Data extraction

The following data were extracted from eligible full publications: (1) study settings including institution and country of origin, (2) study design, (3) study group characteristics, (3) surgical robot details, (4) effectiveness outcomes including biopsy yield and accuracy, and (5) safety outcomes including haemorrhage, transient or permanent worsening neurological deficits, and mortality. Corresponding authors were contacted to provide supplemental data when required.

### Appraisal of evidence

The Methodological Index for Non-Randomised Studies (MINORS) and Jadad scoring systems were undertaken by two researchers independently (VNV and HJM) and used to appraise non-randomised and randomised studies respectively [7, 8]. Correlation between the scores attributed to the studies was calculated using Cronbach’s *α*. Studies of higher quality were given greater weighting in the qualitative review. A pooled analysis of the diagnostic biopsy rate was undertaken with weightings determined by the number of patients in each study. Statistical analysis was performed using SPSS v 24.0 (IBM, IL, USA) and Stata v14 (Statacorp, TX, USA).

### Progressive scholarly acceptance

A bibliometric analysis was performed to determine the extent to which the scientific community has accepted robot-assisted stereotactic brain biopsy. All studies were coded as either an initial investigation or refining study according to the criteria outlined by Schnurman et al. [9, 10]. A compounding model was then used to determine the progressive scholarly acceptance end-point whereby the number of refining studies surpasses the number of initial investigations, implying that the scientific community has accepted the initial questions of effectiveness and safety; some innovations rapidly achieve this transition whilst others fail to ever do so.

## Results

### Summary of search strategy, types of study, and quality of evidence

**Fig. 1:**
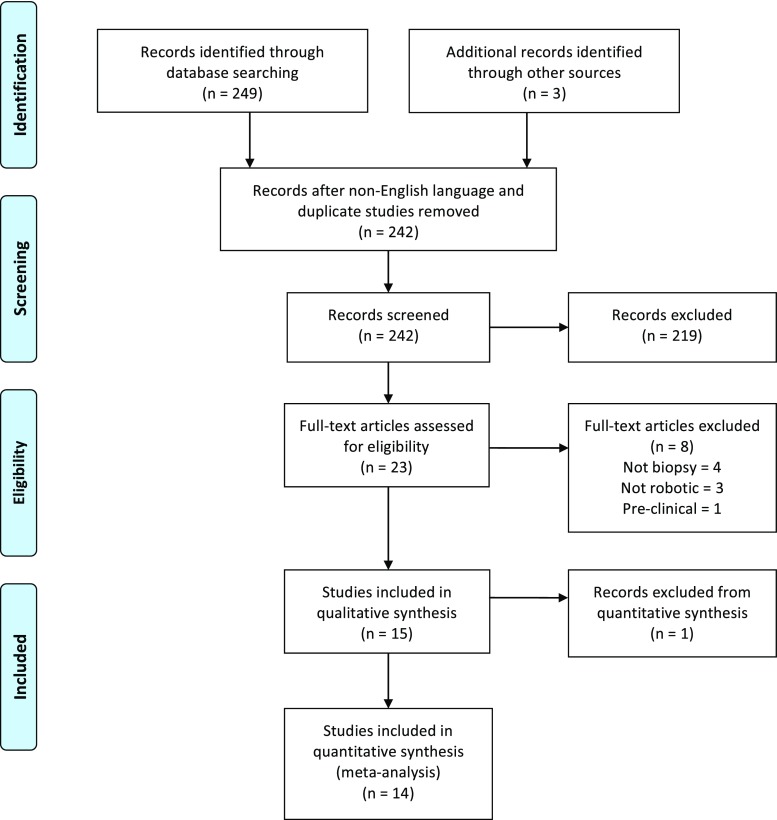
PRISMA flow diagram outlining the study selection process. *From*: Moher D, Llberati A, Tetzlatf J, Altman DG, The PRISMA Group (2009). *Preferred Reporting items for Systematic Reviews and Meta-Analyses*: The PRISMA Statement. Plos Med 6(7): e1000097. 10.1371/journal.pmed1000097. For more information, visit http://www.prisma-statement.org

The database search returned 249 results of which 242 were English language. Screening titles and abstracts identified 24 publications for full manuscript review (Fig. 1). A comparison of the articles identified between the two independent researchers revealed high concordance between included studies. In all, 15 studies were included in the systematic review, comprising a total of 322 patients that underwent 328 robot-assisted brain biopsies (Table 1). As only four studies provided accuracy data and there was no consistent reporting, we were unable to perform a meta-analysis.

**Table 1:** Summary of selected manuscripts reporting robot-assisted brain biopsy. “Pt” means patient. “Not reported” signifies that the authors do not make mention of this in the manuscript, whilst “None reported” signifies that the authors state this outcome did not occur within the study

Author (year) City/state, country	Study design (level of evidence*)	Number of patients Age and sex	Robot	Diagnostic biopsy rate	Accuracy	Complications	Other
Kwoh (1988) California, USA	Case report (level 4)	1 pt 52-year-old male	Unimation Puma 200 robot	1/1 (100%)	None reported	None reported	
Glauser (1995) Lausanne, Switzerland	Case series (level 4)	8 pts	Minerva robot	6/8 (75%)	0.1–0.5 mm	None reported	Use of robot aborted in one operation owing to geometric inaccessibility
Willems (2003) Utrecht, the Netherlands	Case series (level 4)	23 pts Median 53 years (range 22–74 years) 14 female and 9 male	MKM robot with instrument holder	22/23 (96%)	3.3 ± 1.7 mm using bone screws 4.5 ± 2 mm using adhesive markers	2 pts with haematoma: 1 pt asymptomatic and 1 pt with transient worsening of neurological symptoms 1 pt with permanent worsening of neurological symptoms	
Haegelen (2010) Lille, France	Case series (level 4)	15 pts 5 children and 10 adults	Neuromate robot	15/17 (88%)	Not reported	3 pts with neurological symptoms: 2 pts transient and 1 permanent	2 pts had repeat biopsies
Bekelis (2012) New Hampshire, USA	Case series (level 4)	41 pts Mean 60 years (range 33–87 years) 20 male and 21 female	SurgiScope robot	44/45 (98%)	Not reported	5 pts with haematoma: 4 pts asymptomatic and 1 pt that required craniotomy	4 pts had repeat biopsies
Dellaretti (2012) Lille, France	Cohort study (level 4)	33 pts	Neuromate robot	Not reported	Not reported	Not reported	Cohort compared 123 transfrontal and 19 transcerebellar approaches with no significant difference found
LeFranc (2015) Amiens, France	Case series (level 4)	100 pts Median 59 years (range 7–86 years) 67 male and 33 female	ROSA robot	97/100 (97%)	Not reported	6 pts with haematoma: 4 pts asymptomatic and 2 pts with transient neurological symptoms 6 pts with transient worsening of neurological symptoms	
Grimm (2015) Tuebingen and Mainz, Germany	Case series (level 4)	37 pts Range 15–83 years 20 male and 17 female	Renaissance robot	33/37 (89%)	Not reported	9 pts with haematoma: 8 pts asymptomatic and 1 pt that required craniotomy	
Coca (2016) Strasbourg, France	Case series (level 4)	5 pts Mean 9 years (range 5–13 years) 3 male and 2 female	ROSA robot	5/5 (100%)	Not reported	1 pt with transient perioperative bradycardia	
Carai (2017) Pavia, Italy	Case series (level 4)	7 pts Range 5–13 years 5 female and 2 male	ROSA robot	7/7 (100%)	Considered accurate but quantitative data not reported	2 pts with transient worsening of neurological symptoms	
De Benedictis (2017) Parma, Italy	Case series (level 4)	26 pts	ROSA robot	25/26 (96%)	Considered accurate but quantitative data not reported	2 pts with transient worsening of neurological symptoms	
Quick-Weller (2017) Frankfurt, Germany	Case series (level 4)	2 pts 4-year-old male and 12-year-old female	ROSA robot	2/2 (100%)	Not reported	None reported	
Miller (2017) Missouri, USA	Case series (level 4)	6 pts Average age 13 years	ROSA robot	6/6 (100%)	Not reported	None reported	
Minchev (2017) Vienna, Austria	Case series (level 4)	17 pts	iSYS1	16/17 (94%)	Entry point error: median 1.3 mm (range 0.2–2.6 mm) Target point error: median 0.9 mm (range 0.0–3.1 mm)	None reported	Use of robot aborted in one operation as error in image registration
Dlaka (2017) Zagreb, Croatia	Case report (level 4)	1 pt 45 years old	RONNA G3	1/1 (100%)	Entry point error: 2.2 mm Target point error: 2.3 mm	None reported	

*Oxford Centre for Evidence-based Medicine – Levels of Evidence (2009)

All included studies were non-randomised in design including 1 retrospective cohort study and 14 case series or reports (level 4 evidence) [11]. The MINORS system was used to evaluate the quality of these studies, with a high concordance of calculated scores (*α* = 0.98). Studies were of variable quality but few were prospective and none prospectively performed a power calculation for sample size (Table 2).

**Table 2:** Quality of studies using MINORS criteria. The items are scored 0 (not reported), 1 (reported but inadequate) or 2 (reported and adequate). Raw data is displayed for Reviewer 1. “*Pt*” patient, “*NA*” not applicable

Author (year)	Aim	Consecutive pts	Prospective	Outcome	Unbiased evaluation	Follow-up period	Loss to follow-up < 5%	Prospective calculation of study size	Control group	Contemporary group	Baseline equivalence	Statistics	Rater 1	Rater 2	Max score
Case series															
Kwoh (1988)	2	0	2	1	1	2	2	0	NA	NA	NA	NA	10	10	16
Glauser (1995)	2	0	2	1	1	2	2	0	NA	NA	NA	NA	10	11	16
Willems (2003)	2	1	2	2	1	2	2	0	NA	NA	NA	NA	12	12	16
Haegelen (2010)	2	2	0	1	1	2	2	0	NA	NA	NA	NA	10	10	16
Bekelis (2012)	2	1	0	1	1	2	2	0	NA	NA	NA	NA	9	9	16
LeFranc (2015)	2	0	0	1	1	2	2	0	NA	NA	NA	NA	8	8	16
Grimm (2015)	2	2	0	1	1	2	2	0	NA	NA	NA	NA	10	10	16
Coca (2016)	2	2	0	1	1	2	2	0	NA	NA	NA	NA	10	10	16
Carai (2017)	2	2	1	1	1	2	2	0	NA	NA	NA	NA	11	11	16
De Benedictis (2017)	2	1	1	1	1	2	2	0	NA	NA	NA	NA	10	10	16
Quick-Weller (2017)	2	1	0	1	1	2	2	0	NA	NA	NA	NA	9	8	16
Miller (2017)	2	0	0	1	1	2	2	0	NA	NA	NA	NA	8	8	16
Minchev (2017)	2	2	2	2	1	2	2	0	NA	NA	NA	NA	13	13	16
Dlaka (2017)	2	0	1	2	1	2	2	0	NA	NA	NA	NA	10	10	16
Cohort studies															
Dellaretti (2012)	2	0	0	1	1	2	2	0	2	2	1	2	15	14	24

Dellaretti et al. reported a retrospective cohort comparing transcortical and transcerebellar approaches in 142 patients that underwent brain biopsy, of which 33 patients underwent a robot-assisted brain biopsy [12]. Unfortunately, the clinical outcomes of this subset of patients were not separately reported and they have therefore not been included in the pooled analysis of effectiveness and safety.

### Surgical robots

Six studies reported use of the ROSA robot [13–18] and two the Neuromate robot [12, 19]; the other robots used were the Puma 200 robot [2], Renaissance robot [20], Minerva robot (Swiss Federal Institute of Technology of Lausanne, Switzerland) [21], MKM robot (Zeiss, Oberkochen, Germany) [22], Surgiscope robot (ISIS, Grenoble, France) [23], iSYS1 robot (iSYS, Kitzbühel, Austria) [24], and RONNA G3 robot (University of Zagreb, Croatia) [25]. The size and configuration of the reported devices varied significantly with the ROSA and Neuromate robots, occupying a large footprint and weighing up to 200 kg, and the Renaissance and iSYS1 robots being small enough to fix directly to the patient’s head and Mayfield clamp respectively, and weighing as little as 1.4 kg [20, 24].

All robots had a supervisory-controlled function in which the surgeon planned a safe surgical trajectory using pre-operative volumetric imaging, image registration was performed using frame-based or frameless methods, and the robot then carried out trajectory alignment autonomously under the supervision of the surgeon [26]. This trajectory was then used by the surgeon to perform the incision, burr hole craniostomy, and biopsy.

### Effectiveness

The diagnostic biopsy rate varied from 75 to 100% in individual series. Weighted averages based on the number of patients in each study revealed a pooled diagnostic biopsy rate of 94.9% (280/295).

Accuracy measures were provided in six studies, and quantitative data provided in four studies. Within these studies, accuracy measures varied and included Euclidean distance and lateral deviation, or were not specified. When reported, the average target accuracy varied from 0.9 to 4.5 mm.

In the majority of studies, the registration method was not specified. Willems et al. compared the target accuracy of the MKM robot using different registration methods and found that bone-anchored fiducials resulted in significantly greater accuracy than adhesive scalp markers (3.3 ± 1.7 versus 4.5 ± 2 mm) [22].

Only two studies provided accuracy measures for the entry and target point separately. Minchev et al. reporting on use of the iSYS1 robot in 25 patients calculated a median entry point accuracy of 1.3 mm (range 0.2–2.6 mm) and a median target point accuracy of 0.9 mm (range 0.0–3.1 mm) [24]. Dlaka et al. reporting on the RONNA G3 robot in a single patient calculated an entry point error of 2.2 mm and target point error of 2.3 mm [25].

### Safety

Use of the robot was aborted in two operations owing to geometric inaccessibility and an error in image registration but no associated adverse events were reported.

Safety measures reported included haemorrhage rate, transient or permanent worsening neurological deficits, and mortality rate. Post-operative haematoma was reported in 7.5% (22/295) but it is unclear how many patients underwent a routine post-operative scan and only 0.7% (2/295) developed a symptomatic haemorrhage that required craniotomy and evacuation of the haematoma. Neurological deficits occurred in 5.1% (15/295) and permanent neurological deficits in 0.7% (2/295). No mortalities were reported in the pooled analysis.

### Progressive scholarly acceptance

**Fig. 2:**
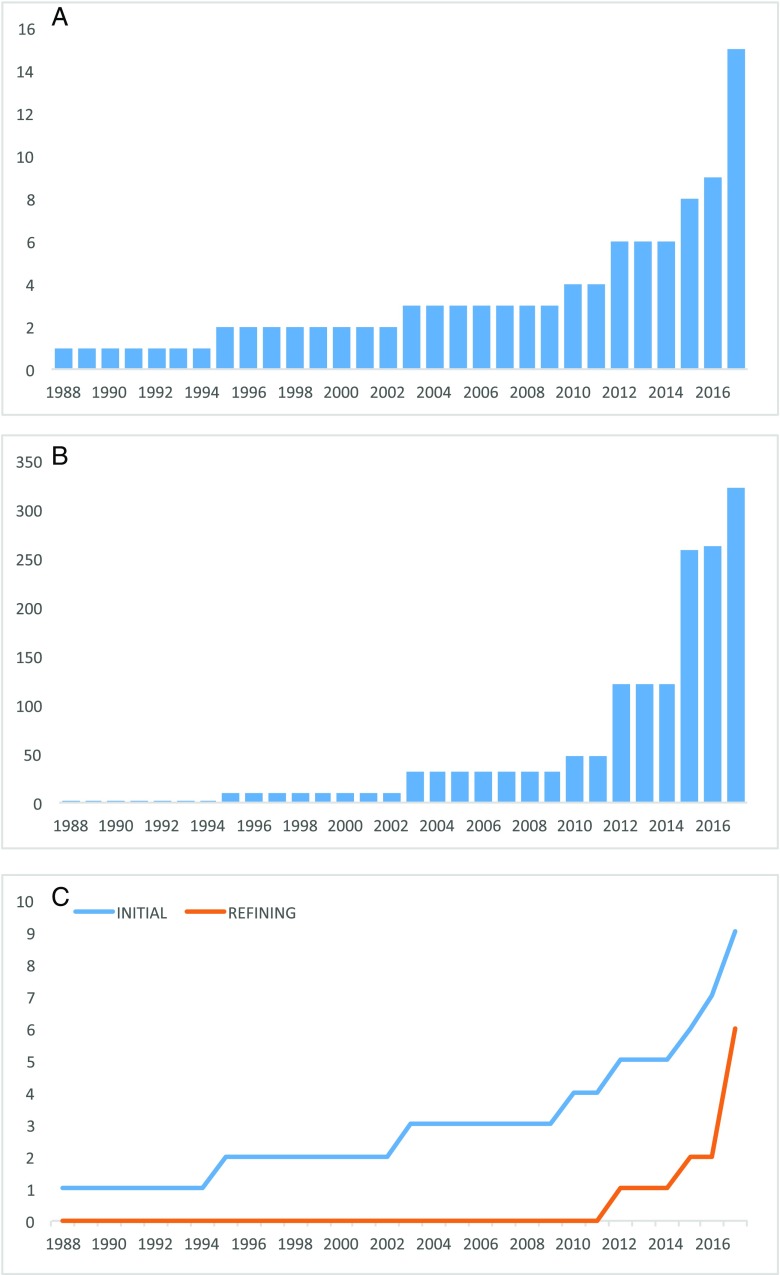
Graphs demonstrating **a** the number of overall publications per annum, **b** the number of patients reported undergoing robot-assisted biopsy per annum, and **c** the number of initial and refining publications per annum

There was a trend towards an increasing number of publications per annum and an increasing volume of procedures per annum (Fig. 2a, b). A compounding progressive scholarly acceptance analysis suggested an early convergence pattern (Fig. 2c), indicating a trend towards acceptance of the technique by the scientific community.

## Discussion

### Summary of evidence

Since the first published report 30 years ago, over 300 patients have undergone robot-assisted brain biopsy. In our pooled analysis, surgery was effective with 95% of procedures resulting in a diagnosis. Although use of the robot had to be aborted in two procedures due to technical errors, there were no associated adverse events as a result of this, and less than 1% of patients had a significant post-operative haematoma or permanent neurological deficit. These findings are further supported by our bibliometric analysis using the progressive scholarly acceptance model, which suggests that the scientific community has begun to accept robot-assisted brain biopsy.

Early surgical robots were modified industrial robots and were large, complex, and expensive. A recent trend towards smaller, simpler, and less expensive platforms has corresponded to their increased adoption within neurosurgery, and the number of procedures that utilises them has grown [27]. These include conventional stereotactic procedures that mandate high accuracy and precision, and have previously relied on frame-based techniques, such as deep brain stimulation [28], stereoelectroencephalography (SEEG) [20], and intracranial catheter placement [24].

Historically, many surgical device innovations have been adopted with little or no evidence to support their effectiveness and safety. In the USA, the majority of such devices are cleared through the 510(k) pathway, which does not require clinical studies [29]. The introduction of devices following clearance is unstructured and variable; more often than not, their use is reported in non-comparative trials without institutional board review. This process carries an obvious risk to patient safety, and a number concerns have been raised regarding the lack of centralised adverse event reporting [30]. To address this shortfall, the Balliol Collaboration has proposed the IDEAL model for safe innovation [3–5]. To this end, our bibliographic analysis suggests that robot-assisted brain biopsy currently lies in Phase 2b (Exploration) and that research databases and comparative trials are now warranted (Table 3).

**Table 3:** Defining characteristics of phases of surgical and interventional innovations (adapted from http://www.ideal-collaboration.net/about-ideal/ideal-summary-tables/)

	1. Idea	2a. Development	2b. Exploration	3. Assessment	4. Long-term monitoring
Purpose	Proof of concept	Development	Learning	Assessment	Surveillance
Number and types of patients	Single digit; highly selected	Few; selected	Many; may expand indications	Many; expanded indications	All eligible
Number and types of surgeons	Very few	Few; innovators and some early adopters	Many; innovators, early adopters, early majority	Many; early majority	All eligible
Study types	Structured case reports	Prospective development studies	Research databases; feasibility RCT	Surgical randomised controlled studies	Prospective registries

### Comparison with other studies

To our knowledge, there have been no previous reviews evaluating the accuracy, effectiveness, and safety of robot-assisted brain biopsy. However, our pooled analyses of outcome data are comparable with previously reported non-robotic frame-based and frameless biopsy series [31].

Khatab et al. reviewed 16 studies in which 1628 frameless brain biopsies were performed using optical guidance, and found an average diagnostic yield of 93.8% [32]. Similarly, Frati et al. reported on 296 cases over an 8-year period from a single institution that underwent frameless biopsy with a diagnostic yield of 99.7% [33]. Harrisson et al. reported pinless frameless biopsy in 149 patients with electromagnetic guidance and found that 5 cases were non diagnostic, although in 4 of these cases, the specimen was of abnormal tissue but the pathologist was unable to make a diagnosis [34]. This highlights an important limitation in using diagnostic biopsy rate as an outcome measure as newer genetic markers may improve the diagnostic rates in the future.

Few studies have reported the accuracy of brain biopsy. In a single-centre randomised controlled trial, Bradac et al. compared frame-based and frameless brain biopsy in 53 patients and calculated a target point accuracy of 2.65 ± 1.12 and 2.90 ± 1.26 mm respectively [35].

Kulkarni et al. investigated the rate of haemorrhages following non-robotic stereotactic biopsy in 102 patients based on post-operative CT scans [36]. This revealed 59.8% (61/102) patients developed haemorrhages of which 54.9% (56/102) were intracerebral. The incidence of clinically significant symptomatic haemorrhage was 5.8% (6/102) whilst the remaining 53.9% (55/102) were clinically silent. This raises the question of whether clinically silent haemorrhages are a useful outcome measure as they ultimately have no effect on the patient. Further, it is likely that had patients undergone post-biopsy MRI sequences that are more sensitive to blood products, such as susceptibility-weighted imaging, an even greater incidence would have been detected.

### Limitations

The present review was restricted to relatively few studies, of variable size and quality, and with inconsistent reporting of surgical outcomes, which necessarily limits the conclusions that can be drawn. This finding is consistent with an innovation in the early adoption phase [37].

In practice, the accuracy and precision of surgical robots depends on a number of factors. Arguably, the most important of these is the registration of the patient to the pre-operative reference imaging, upon which the biopsy trajectory has been planned. Image registration can be in the form of fiducial markers, with Willems et al.’s finding that bone-anchored fiducials resulted in significantly greater accuracy than adhesive scalp markers [22]. The use of intra-operative imaging allows automatic registration methods with greater accuracy than scalp and bone-anchored fiducials [38].

Other factors that may influence surgical outcome of robot-assisted brain biopsy include the location of the lesion, the planned trajectory, and the histological nature of the lesion. Dellaretti et al. compared transcortical and transcerebellar approaches in patients undergoing brainstem biopsy and did not find any significant difference in outcomes [12]. Nonetheless, given that there is no systematic or objective means of trajectory planning at present, this remains a significant confounding factor.

Careful trajectory planning is critical to avoiding complications in stereotactic procedures. In stereotactic brain biopsy procedures that utilise a burr hole craniostomy for trajectory determination, it is not possible to visualise the cortical vasculature. The safety of such procedures is therefore dependent on the ability of pre-operative imaging to visualise cerebral vasculature so that planned trajectories avoid this along their entire length. Avoiding sulcal-pial boundaries, reducing intracerebral length, and orthogonal traversing of the skull have all been suggested to improve safety of stereotactic procedures [39]. Computer-assisted algorithms for trajectory planning have been shown to optimise these factors in a systematic fashion for DBS and SEEG, but there are no studies that have utilised this for stereotactic biopsy planning [39, 40].

## Conclusions

Robot-assisted stereotactic brain biopsy is an increasingly mainstream tool in the neurosurgical armamentarium. Although limited, the literature suggests the technique is as effective and safe as the existing frame-based and frameless biopsy. Moreover, our bibliometric analysis suggests that the scientific community has begun to accept robot-assisted brain biopsy. Further evaluation should proceed along the IDEAL framework with research databases and comparative trials.
